# Supplementary material for: Prediction and visualization of Mergers and Acquisitions using Economic Complexity
Source: PLoS One. 2023 Apr 3;18(4):e0283217. doi: 10.1371/journal.pone.0283217 (PMC10069768; doi:10.1371/journal.pone.0283217)
Supplement: S1 File — (PDF) [file pone.0283217.s001.pdf]

Supplementary Information

Prediction and visualization of Mergers and Acquisitions using Economic Complexity

Lorenzo Arsini, Matteo Straccamore and Andrea Zaccaria

Contents

1 **Pair Prediction on the full database** 3

2 **Evaluation metrics robustness test** 3

3 **Predictions in different industrial sectors** 4

4 **Coherence γ and RF VS diversification** 5

5 **Optimisation of α in CCS Jaffe + Sector** 6

6 **Crunchbase name matching** 7

7 **Sectors Classification** 8

1 Pair Prediction on the full database

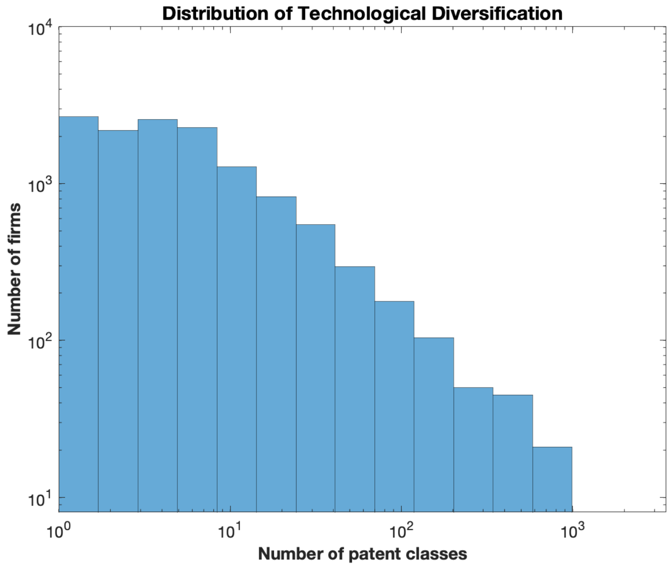

Figure 1: **Distribution of technological diversification.** The diversification of companies in our database follows a power-like distribution, where low diversified firms are prevalent, and this implies that, if the negative examples companies are drawn from the entire database, using metrics that are correlated with diversification leads to better predictions.

As stated in the Result section, the performances of some measures employed in this work, in the Pair Prediction task, can depend on the diversification of companies chosen for the negative examples. This is the case of indirect measures based on networks and Random Forest and direct measures based on networks. The diversification of companies in our database follows a power-like distribution, where low diversified firms are prevalent (see Figure 1). This implies that, if the negative examples companies are drawn from the entire database, using metrics which is correlated with diversification leads to better predictions. In Figure 2 we present the Best F1 values relative to the Pair Prediction task in this case. Note that with these conditions, it was not possible to construct the Jaffe + Sectors measure and the relative CCS because we don't have information on the industrial Sector of all companies. As it is possible to see in Figure 2, all the rescaled metrics perform worse than the original ones. This is true also for the Jaffe - Common Tech pair of measures in facts, now the best-performing measure for predictions is Common Tech. Note that in any case, we found that a direct measure based on an angular distance between companies in the technology space is better than any other direct or indirect metric employed. As a final remark we can note that independently from the relative Best F1 levels among the various measures, predictions done using negative examples from the whole database are much better than the ones presented in the Result section, done extracting the negative samples from a set of companies whose industrial sectors are known. This demonstrates, in general, that the choice of negative examples strongly influences the power of our predictions.

2 Evaluation metrics robustness test

In this Section we reproduce the results of Figure 2 in the main text, using different evaluation metrics. The metrics we decided to use are:

- Area Under Precision-Recall Curve (PRC-AUC) [1]: the area under the curve in the precision-recall plane. The latter quantities are obtained by varying the threshold that identifies the value above which the scores are associated with positive predictions. We decide to calculate it normalizing by the random case in the same way as the work of Futagami et al. [2].

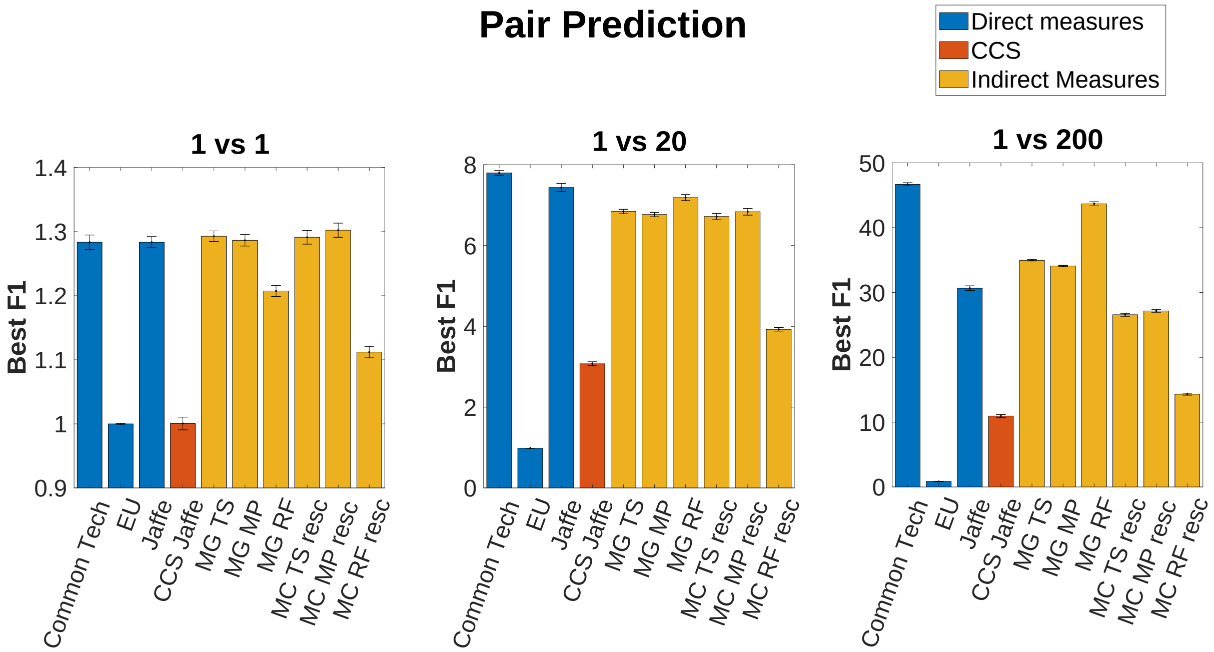

Figure 2: **Comparison between Best F1 on Pair Prediction.** We show the comparison between our defined similarity metrics. We chose as values of Class Imbalance: 1 VS 1, 1 VS 20, and 1 VS 200, which means that for each true M&A, we extract 1, 20, and 200 false negatives, i.e. 1, 20, or 200 not real M&A. In both figures, we use three different colors for each different category's metrics. Blue represents the direct metrics, yellow the indirect ones and orange the CCS ones. Error bars are present because, for each class imbalance, we repeat the exercise of selecting random Target companies and/or Acquirer companies 20 times, calculating the mean and standard deviation of the mean. In this case, it was not possible to construct the Jaffe + Sectors measure and the relative CCS because we don't have information on the industrial Sector of all companies. In all panels, Best-F1 values are normalised to ones of random predictions. With this normalisation, a Best-F1 value around 1 will indicate a random-like prediction, while values greater than 1 will correspond to more significant predictions

- Precision at 1000: the fraction of the largest 1000 elements of the score predictions that are actually true.

The results, Figure 3, are consistent with those in the main text using the Best-F1 metric with the Jaffe + Sector which is the best prediction algorithm. About the best α for both Jaffe + Sector and CCS J + S, the best one is the same as the Best-F1 metric.

3 Predictions in different industrial sectors

In this section, we investigate how our predictions vary across different industrial sectors. In order to do this, we divided our M&As according to the acquirers' industrial sector, using the classification employed in the main text and explained in Section 7 of the SI. For each acquisition, we computed our prediction score using our best-performing measure: Jaffe + Sectors. We are also considering only the Target Prediction task, because we are fixing the acquirer and its industrial sector, and we chose an intermediate class imbalance of 1 vs 20: for each true M&A, 20 random targets are randomly extracted for the same acquirer as negative cases. Results are presented in Figure 4. It is interesting to note how the technological relatedness, calculated with our measure, has high predictive power in most of the sectors and in particular in the ones that are prone to research and innovation (for example Health Care, Medical Research, Services, and Security). On the other hand, the occurrence of M&As in the "Economy and Finance" sector seems not to be correlated with the similarity between companies at the patent level. From our point of view, such a result has a clear interpretation. Such companies mostly belong to Financial Services and Investments sectors. In these sectors M&As are most likely done, not looking for similar innovative activities, but

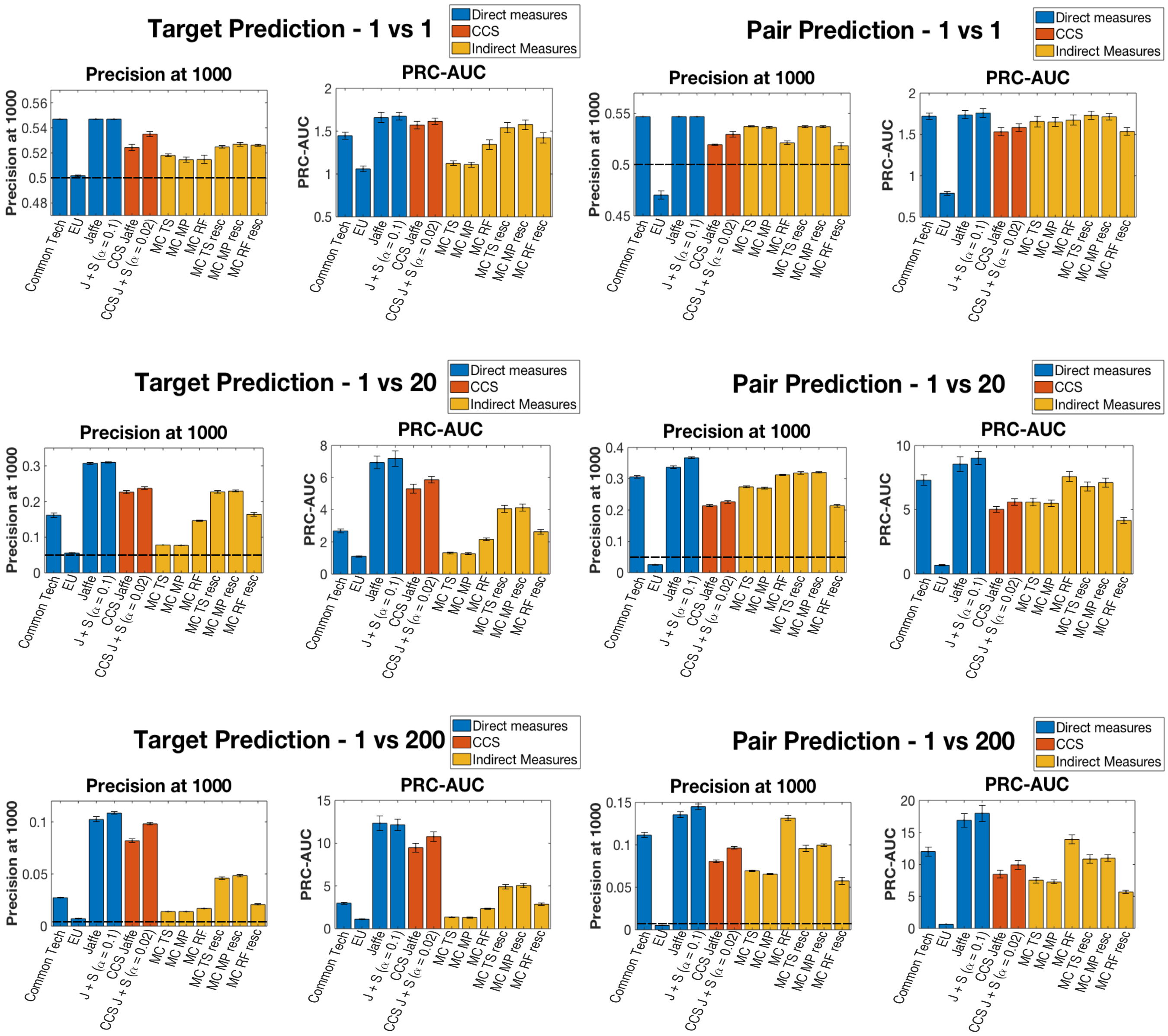

Figure 3: **Predictions evaluation of Target and Pair forecasts for other metrics.** In all figures, we show the comparison between our defined similarity metrics. We chose as values of Class Imbalance: 1 VS 1, 1 VS 20, and 1 VS 200, which means that for each true M&A, we extract 1, 20, and 200 false negatives, i.e. 1, 20, or 200 not real M&A. In all figures, we use three different colors for each different category's metrics. Blue represents the direct metrics, yellow the indirect ones and orange the CCS ones. Error bars are present because, for each class imbalance, we repeat the exercise of selecting random Target companies and/or Acquirer companies 20 times, calculating the mean and standard deviation of the mean. In the Precision at 1000 panels the black dashed lines represent the levels relative to a random prediction.

rather seeking the best investments in different areas, thus they cannot be predicted based on technological affinity.

4 Coherence γ and RF VS diversification

In the main text, we explain the necessity to rescale the Indirect measure. The reason is that γ and RF are highly correlated with diversification. We can see these correlations in Figure 5**a-c**. Rescaling between 0 and 1 these measures, we can partially remove that dependence (as seen in Figure 5**b-d**) and quantify its effects on the forecasts.

To quantify the correlation between these quantities we compute the absolute value of the spearman correlation, equal to 0.90 for γ and 0.69 for RF. After the rescaling process, in **b** and **d** we show the new correlation, with a spearman correlation of 0.65 for γ and 0.42 for RF. The rescaling can lower the

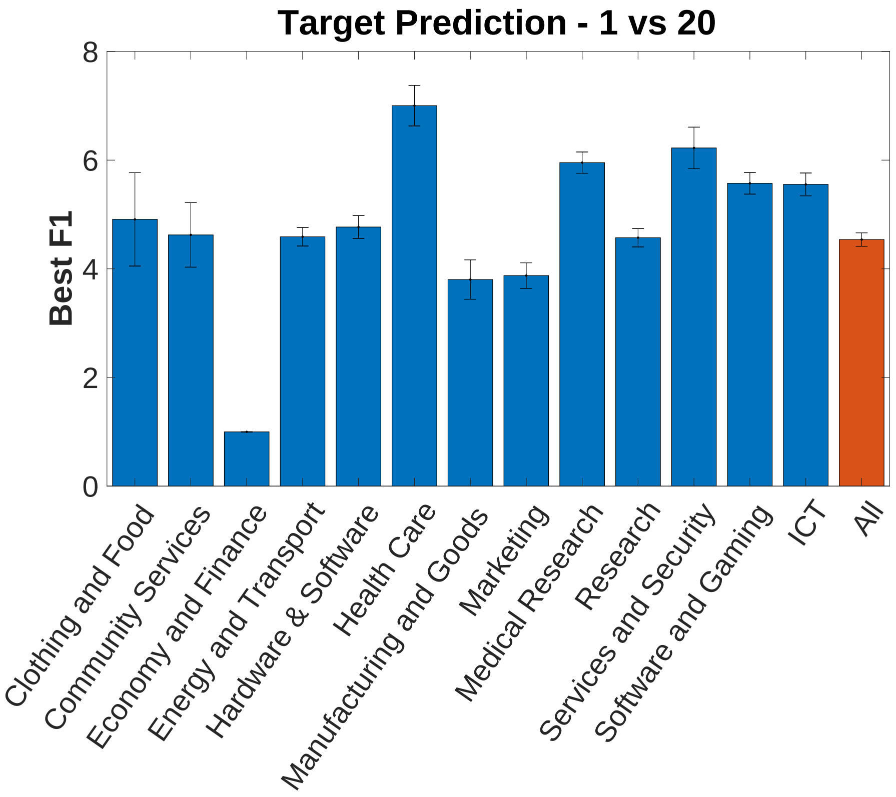

Figure 4: **Comparison between Best F1 on Target Prediction with class imbalance 1 vs 20 for different industrial sectors.** M&As were divided on the basis of the acquirers' industrial sectors and evaluated using our best-performing measure: Jaffe + Sectors. Bars report the Best-F1 values normalised to the random prediction case. Results show how our measure has great predictive power, especially in sectors where research and innovation are crucial. On the other hand, in the "Economy and Finance" sector, where M&As are most likely done as investments and not looking for innovation in the same field, a technological relatedness measure has low predictive power.

correlation between the two quantities, and the effects of this can be seen in the main text Results.

5 Optimisation of α in CCS Jaffe + Sector

To find the best α parameter for the CCS measure, we do the same computations explained in the Methods Section main text in the Jaffe case. Unlike the optimization of α over Jaffe, in the case of CCS we notice that starting from relatively low values of class imbalance (1VS5, for example), the addition of industry information leads to lower forecast results. In other words, starting from such values of class imbalance the optimal α is close to 0. In Figure 6 we show the heat map of the Best F1 as a function of α and the class imbalance, both for the Pair Prediction and the Target Prediction. To average out some fluctuations coming from the random extraction of negative examples, each point in the Figures reports the mean Best F1 over 20 realizations of the predictions. Because the Best F1 is highly correlated with the class imbalance, in these Figures, for each value of N we rescaled the Best F1 between 0 and 1. In this way, it is possible to better spot the maximum of Best F1 as a function of α, for each value of class imbalance. As it is possible to extrapolate from the figures, if we increase the class imbalance, the maximum of Best F1 remains around low α values for different values of class imbalance, becoming increasingly evident as class imbalance increases. This suggests that when choosing a M&A pair among a large pool of options, the similarity between the companies from the point of view of the industrial sector becomes increasingly less important in contrast to a technological similarity.

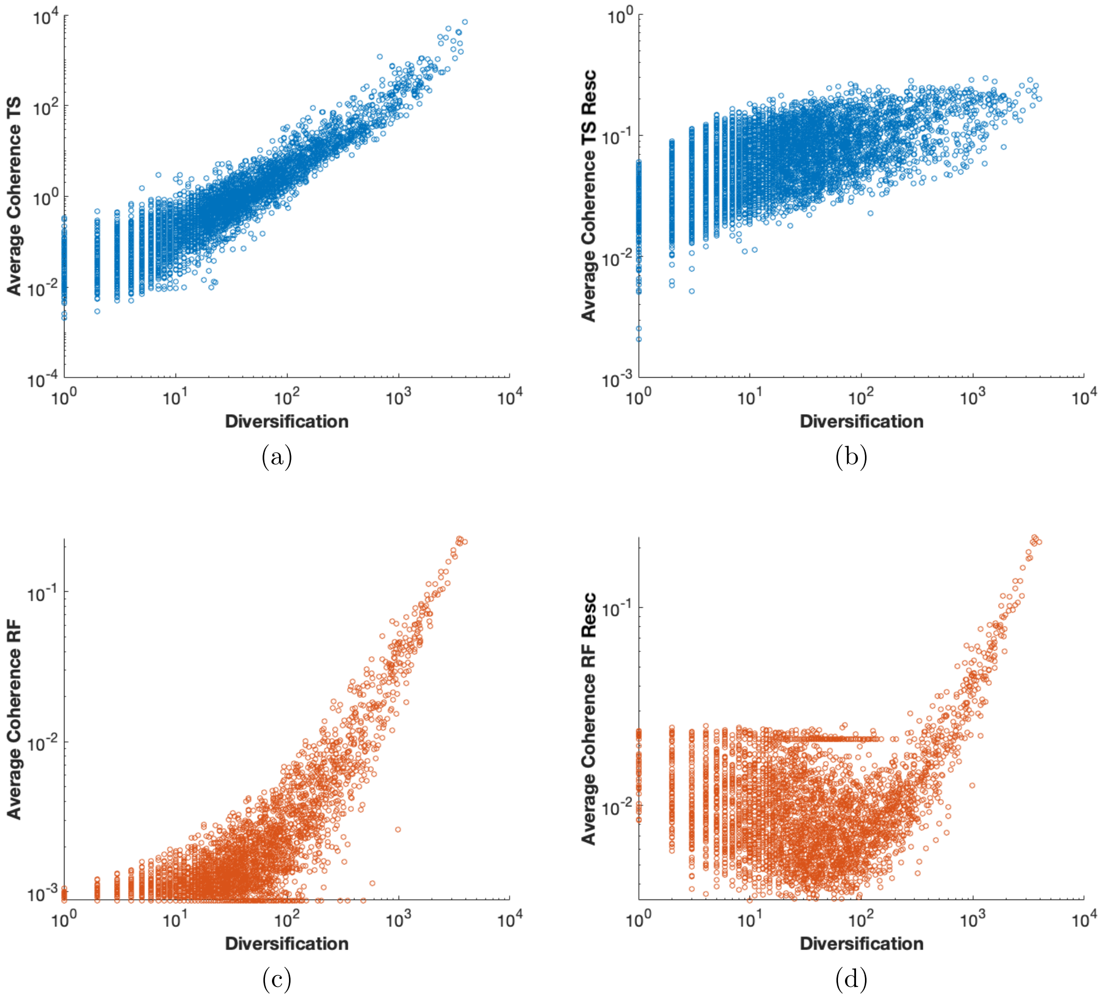

Figure 5: **Indirect measures VS diversification: before and after rescaling** In **a** and **c** we show the correlation between the Coherence γ and RF, and the diversification before the rescaling process. To quantify the correlation between these quantities, we compute the spearman correlation, equal to 0.90 for γ and 0.69 for RF. After the rescaling process, in **b** and **d** we show the new correlation, with a spearman correlation of 0.65 for γ and 0.42 for RF. The rescaling can lower the correlation between the two quantities, and the effects of this can be seen in the main text Results.

6 Crunchbase name matching

To associate Cruchbase companies with BVDIDs and then with their technological portfolios, it was necessary to match their names to those contained within AMADEUS, in which the BVDID of the companies are reported. To get a good match between the names, these were "cleaned" and standardized, performing the removal of symbols, special characters, spaces, and company acronyms (S.p.a, srl, etc.). For this last task, the *cleanco* package for Python was used. In general, in the names cleaning process, we followed these steps:

1. Conversion of names to uppercase, removal of commas, dashes, and text between parentheses.
2. First execution of *cleanco*, dots removal and second execution of *cleanco*, removal of European nations' names.
3. Removal of all remaining symbols, special characters, and spaces.

After the cleaning, the names match was performed for string equality. To limit the errors due to poor or excessive cleaning of names, the match was performed 3 times with 3 different levels of cleaning and the results were then merged. The 3 cleaning levels differ in the partial or total execution of the second step: in the first level *cleanco* is executed only once, in the second level the second execution of *cleanco* takes place after the removal of dots and, finally, in the third level names of the European nations are removed.

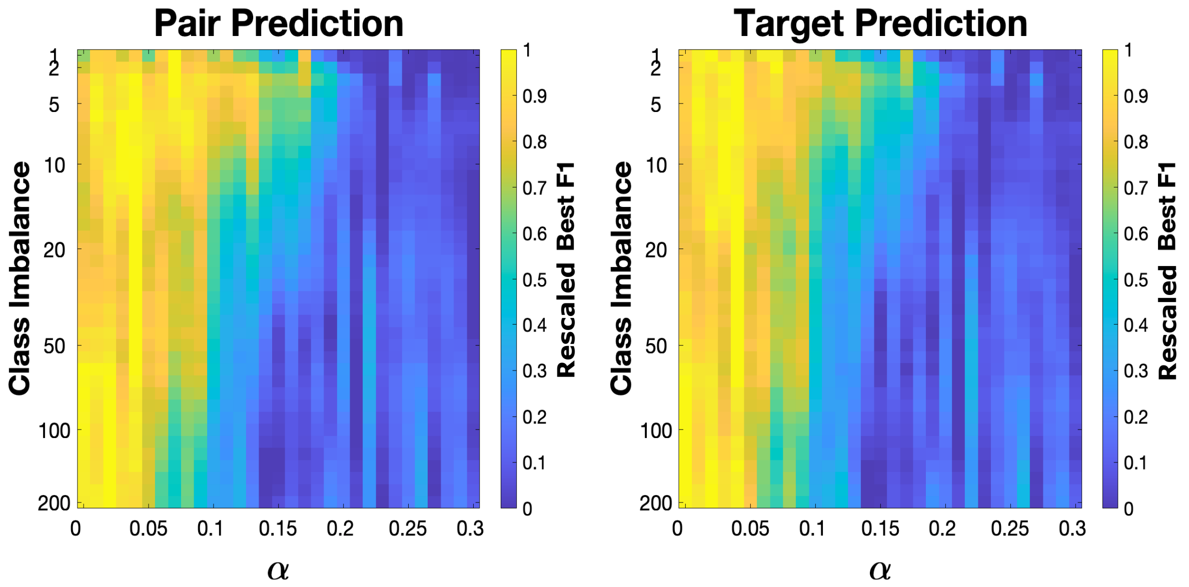

Figure 6: **Dependence of maximum Best F1 on α and Class imbalance in CCS Jaffe + Sectors model**. We show the behavior of the Best F1 for α and the class imbalance. For each value of class imbalance, we rescale the Best F1 between 0 and 1 to better see the maximum as a function of α. The Best F1's maximum remains around low α values for different values of class imbalance, and this becomes more evident more the class imbalance grows. This suggests that when choosing a M&A pair among a large pool of options, the similarity between the companies from the point of view of the industrial sector becomes increasingly less important in contrast to a technological similarity.

In this way, it was possible to associate at least a BVDID to 28137 companies out of the 123576 in the Crunchbase database and involved in acquisitions.

7 Sectors Classification

The companies in the Crunchbase database are organized, according to the industrial sector, on two levels of aggregation: the lowest counts 744 *categories*, while the highest counts 43 *category groups*. The classification is not related to the official ones (NACE NAICS, SIC, etc.), but was built independently by Crunchbase. As it is constructed, this classification is not useful for the purposes of this work because:

- Many *category groups* (and consequently several *categories*) are associated with a single company.
- The *category groups* number is too large for visualisation purposes.

For these reasons, starting from the classification of Crunchbase in *category groups*, we defined a new classification in 13 industrial sectors, identified by the letters from A to M. In this way, we are able to assign a unique industrial sector to about 70% of the companies that we had previously linked to their technological portfolio.

The construction of this classification took place in 4 phases:

- First division of *category groups* into 12 sectors.
- Assignment of sectors A and B *Manufacturing and Goods* and *Hardware & Software* to companies: the associated *category groups* are the most common, therefore only the companies which were assigned exclusively to the respective *category group* have been classified in sectors A and B.
- Assignment of remaining sectors: depending on the associated *category groups* one or more sectors have been assigned to each company.
- Specific reassignments: some companies, linked to two sectors, have been reassigned to only one of the two. In this phase, the thirteenth sector, the *Medical Research* one, was created.

Sectors, associated *category groups*, and subsequent reassignments are listed in Table 1.

Table 1: Industrial sector classification for Crunchbase's companies

Sectors	Label	Reassigned Labels	Category Groups
Manufacturing & Goods	A		Consumer Goods<br>Manufacturing
Hardware & Software	B		Hardware<br>Software
Clothing & Food	C	CG	Agriculture and Farming<br>Clothing and Apparel<br>Food and Beverage
Software & Gaming	D	DF, DL, DE	Apps<br>Consumer Electronics<br>Gaming<br>Mobile<br>Navigation and Mapping<br>Platforms
Research	E		Artificial Intelligence<br>Biotechnology<br>Data and Analytics<br>Science and Engineering
Energy & Transport	F	EF, DF	Energy<br>Natural Resources<br>Sustainability<br>Transportation
Community Services	G		Community and Lifestyle<br>Design<br>Education<br>Events<br>Media and Entertainment<br>Real Estate<br>Sports<br>Travel and Tourism
Services & Security	H	HL	Administrative Services<br>Government and Military<br>Privacy and Security<br>Professional Services
Health Care	I		Health Care
Economy & Finance	J		Financial Services<br>Lending and Investments<br>Payments
Marketing	K	KL, GK	Advertising<br>Commerce and Shopping<br>Content and Publishing<br>Sales and Marketing
ICT	L	EL	Information Technology<br>Internet Services<br>Messaging and Telecommunications<br>Music and Audio<br>Video
Medical Research	M	EI	

References

1. Andreas Beger. Precision-recall curves. 2016.

2. Katsuya Futagami, Yusuke Fukazawa, Nakul Kapoor, and Tomomi Kito. Pairwise acquisition prediction with shap value interpretation. *The Journal of Finance and Data Science*, 7:22–44, 2021.
